# The Role of Mesenchymal Stromal Cells-Derived Small Extracellular Vesicles in Diabetes and Its Chronic Complications

**DOI:** 10.3389/fendo.2021.780974

**Published:** 2021-12-20

**Authors:** Fu-Xing-Zi Li, Xiao Lin, Feng Xu, Su-Kang Shan, Bei Guo, Li-Min Lei, Ming-Hui Zheng, Yi Wang, Qiu-Shuang Xu, Ling-Qing Yuan

**Affiliations:** ^1^ National Clinical Research Center for Metabolic Disease, Department of Endocrinology and Metabolism, The Second Xiangya Hospital, Central South University, Changsha, China; ^2^ Department of Radiology, The Second Xiangya Hospital, Central South University, Changsha, China

**Keywords:** diabetic complication, extracellular vesicles, insulin resistance, mesenchymal stromal cells, microRNAs

## Abstract

Mesenchymal stromal cells (MSCs) are applied in regenerative medicine of several tissues and organs nowadays by virtue of their self-renewal capabilities, multiple differentiation capacity, potent immunomodulatory properties, and their ability to be favourably cultured and manipulated. With the continuous development of “cell-free therapy” research, MSC-derived small extracellular vesicles (MSC-sEVs) have increasingly become a research hotspot in the treatment of various diseases. Small extracellular vesicles (SEVs) are membrane vesicles with diameters of 30 to 150 nm that mediate signal transduction between adjacent or distal cells or organs by delivering non-coding RNA, protein, and DNA. The contents and effects of sEVs vary depending on the properties of the originating cell. In recent years, MSC-sEVs have been found to play an important role in the occurrence and development of diabetes mellitus as a new way of communication between cells. Diabetes mellitus is a common metabolic disease in clinic. Its complications of the heart, brain, kidney, eyes, and peripheral nerves are a serious threat to human health and has been a hot issue for clinicians. MSC-sEVs could be applied to repair or prevent damage from the complications of diabetes mellitus through anti-inflammatory effects, reduction of endoplasmic reticulum-related protein stress, polarization of M2 macrophages, and increasing autophagy. Therefore, we highly recommend that MSC-sEVs-based therapies to treat diabetes mellitus and its chronic complication be further explored. The analysis of the role and molecular mechanisms of MSC-sEVs in diabetes and its related complications will provide new idea and insights for the prevention and treatment of diabetes.

## Introduction

Diabetes is a group of metabolic diseases characterized by chronic hyperglycemia caused by multiple causes. It is caused by defects in insulin secretion and/or function. By 2045, the number of diabetic patients is predicted to rise to 693 million (1). Diabetes is mainly manifested by absolute or relative deficiency of insulin and decreased sensitivity of target cells to insulin (2). Persistent high blood sugar can cause extensive vascular damage to the cardiovascular system, retina, kidneys, and nerves, which can lead to various complications (3–5). At present, the application of therapeutic insulin and oral hypoglycemic agents is one of the methods to effectively control the blood glucose level of diabetic patients (6, 7). However, the long-term use of insulin and hypoglycemic drugs causes side effects of varying degrees, and exogenous insulin is still not enough to mimic the natural activity of endogenous insulin. There is also a risk of hypoglycaemia (8). In addition, transplantation of pancreatic or islet cells has been widely restricted in clinical applications due to the lack of pancreatic donors, the number and activity of pancreatic cells, allogeneic immune rejection, surgery and post-operation and many other complex factors (9).

Mesenchymal stromal cells (MSCs) have been established as promising candidate sources for cell therapy due to their contributions to tissue and organ homeostasis, repair and support by self-renewal and multi-differentiation, as well as by their anti-inflammatory, anti-proliferative, immunomodulatory, trophic and pro-angiogenic properties (10). Various diseases have been successfully treated by MSCs in animal models and hundreds of clinical trials related to the potential benefits of MSCs are in progress or have concluded satisfactorily (11). MSCs are commonly used in hematopoietic stem cell transplantation, repair of tissue injuries (bone, cartilage, joint, heart, liver, spinal cord, and nervous system diseases), autoimmune diseases, and as vectors for gene therapy (12). MSCs can promote the regeneration of pancreatic β-cells, protect endogenous pancreatic β-cells from apoptosis, and improve the insulin resistance (IR) of peripheral tissues by providing a supportive microenvironment driven by the secretion of paracrine factors or the deposition of extracellular matrix (13, 14). Numerous studies have shown that the therapeutic effects of MSCs are mediated in a paracrine manner, mainly through extracellular vesicles such as small extracellular vesicles (sEVs) (15). Therefore, cell-free therapy technology based on MSC-derived small extracellular vesicles (MSC-sEVs) has gradually become a research direction. Existing studies have shown that MSC-sEVs have a therapeutic effect on diabetes (16, 17). MSC-sEVs also show great potential in the tissue repair of diabetes complications. Therefore, the regenerative and immunomodulatory properties of MSC-sEVs have the potential to treat diabetes and related complications, such as diabetic nephropathy (DN) (18) and central nervous system damage (19). Information has emerged regarding the roles of specific miRNAs and other MSC-sEVs components as mediators of the protective effects of MSCs administration in preclinical diabetes disease models but many remains unknown. Currently, the role of MSC-sEVs in the treatment of diabetes diseases is an area of active preclinical study. This review mainly introduces the research progress of MSC-sEVs in the treatment and pathogenesis of diabetic central and peripheral neuropathy, diabetic vascular disease, diabetic skin disease, and DN in recent years.

## Diabetes

Diabetes describes a group of conditions in which blood glucose is not properly regulated. Diabetes mellitus occurs when β-cells fail to secrete the insulin necessary to maintain the homeostasis of glucose in the blood. The most common forms of diabetes are type 1 (T1DM) and type 2 diabetes mellitus (T2DM). T1DM results from a cell-mediated autoimmune destruction of β-cells, whereas in T2DM, IR from peripheral organs is coupled with insulin deficiency resulting from an insufficient β-cell mass or function. Other forms of diabetes include gestational diabetes, latent autoimmune diabetes of adulthood (LADA), and neonatal diabetes mellitus (NMD) and maturity onset diabetes of the young (MODY) in which mutations in key pancreatic genes are found (e.g. Glucokinase, Pdx1, etc.). Over time, diabetes can lead to the development of different long-term complications such as diabetic retinopathy, neuropathy, nephropathy, critical ischemia of the limbs and so on. The pathogenesis of related diabetes complications was shown in **Table 1**. Currently, diabetes cannot be cured, and the treatment of diabetes consists of handling hyperglycemia by providing an exogenous insulin and medications supply or by islet cell transplantation. However, the inability to achieve tight control of glucose regulation has motivated more efforts to develop other approaches to address diabetes and reduce the burden of existing diabetes complications. Moreover, the diabetes-based existence of a chronic inflammatory state, impaired immune response, impaired coagulation and other related complications could be among the underlying pathophysiological mechanisms contributing to the increased morbidity and mortality of people with diabetes.

**Table 1:** Application of MSC-sEVs in diabete diseases.

Types of diabetes complications	Pathogenesis of diabetic complications	MSC-sEVs source	MSC-sEVs dose used	MSC-sEVs delivery	MSC-sEVs Isolation	Mechanisms	Reference
Diabetic autonomic neuropathy	Ischemia, hypoxia, activation of polyol metabolic pathways, reduced inositol synthesis, genetic factors, and autoimmune impairment	Rat AD-MSCs	200 μg/0.1 mL PBS *in vivo*	IV	ExoQuick	Delivering corin, anti-inflammatory	(20)
		Rat AD-MSCs	10-100 μg *in vivo*	IV	ExoQuick	Exosomal miRNA transfer	(21)
		Rat BM-MSCs	100 μg/0.2 mL PBS *in vivo*	IV	UCF	Exosomal miR-21-5p transfer, inhibiting the expression ofPDCD4	(22)
Diabetic retinopathy	Oxidative stress, susceptibility genes, activation of polyol metabolic pathways, role of cytokines, non-enzymatic glycosylation of proteins, activation of protein kinase C	Human BM-MSCs	4 μL of 1 x 10^6^ particle/mL	vitreous humor	ExoQuick	Enhancing functional recovery, reducing neuroinflammation and cell apoptosis	(23)
		Rabbit AD-MSCs	100 mg p/mL	IV	UCF	Exosomal miR-222 transfer	(24)
		Human UC-MSCs	250 μg/mL	*ex vivo*	UCF	Exosomal miR-1 26 transfer, suppressing the HMGBI signaling pathway	(25)
Macrovascular disease	Injury of endothelial cells, proliferation of smooth muscle cells, enhancement of platelet aggregation and adhesion	Rat BM-MSCs	5-20 μg/mL	*ex vivo*	UCF	Exosomal miR-146a transfer to VSMCs	(26)
Diabetic nephropathy	Genetic factors, abnormal renal hemodynamics, metabolic abnormalities caused by hyperglycemia, hypertension, abnormal metabolism of vasoactive substances	Mouse BM-MSCs	100 μg/kg *in vivo*	IV	UCF	Enhancing autophagy through the mTOR signaling pathway	(11)
		Mouse AD-MSCs	25 μg/mL *in vitro*	IV	ExoQuick	Exosomal miR-146a transfer inhibition of Smad1/mTOR signaling pathway in podocyte	(27)
		Rat BM-MSCs	5.3 × 10^7^/0.2 mL PBS *in vivo*	RSI	ExoQuick	Anti-apoptotic effect and protecting tight junction structure in tubular epithelial cells	(28)
		Human USCs	not reported	*ex vivo*	UCF	Exosomal miRNA transfer, mainly miR-145	(29)
		Human USCs	100 μg/0.2 mL PBS *in vivo*	IV	UCF	Reducing the urine volume and urinary microalbumin excretion, preventing podocyte cell apoptosis	(18)
		Human USCs	10 μg /0.2 mL PBS *in vivo*	IV	ExoQu ick	Exosomal miR-16-5p transfer to podocytes	(30)
		Mouse AD-MSCs	not reported	*ex vivo*	ExoQuick	Exosomal miR-215-Sp transfer to podocytes	(31)
		Human UC-MSCs	25, 50, 100 μg/mL *in vitro*	*ex vivo*	Not reported	Depressing cytokine expression	(32)
Diabetic foot ulcer, diabetic skin damage	Neurological and vascular lesions and traumatic infections. Glycoprotein deposition on the basement membrane of capillaries thickens the tube wall and causes hypoxia in tissues, resulting in microvascular lesions	Human BM-MSCs	not reported	SUB	ExoQuick	Anti-inflammatory, increasing ratio ofM2/M1 polarization	(33)
		Human MB-MSCs	10 μg/0.1 mL PBS *in vivo*	SUB	UCF	Inducing Ml/M2 polarization, enhancing neoangiogenesis, activating of the NF-κβ	(34)
		Human GG-MSCs	150 μg/0.1 mL in PBS *in vivo*	hydrogel	UCF	Promoting re-epithelialization, enhancing angiogenesis and neuronal ingrowth.	(35)
		Human UC-MSCs	60 μg/0.5 mL in PBS *in vivo*	SUB	UCF	let-7b, regulating macrophage plasticity through activating TLR4/NF- κβ /STAT3/AKT signaling	(36)
		Human AD-MSCs	not repotted	not repotted	ExoQuick	Overexpressing Nrf2, decreasing ROS, anti-inflammatory	(37)
		Human UC-MSCs	200 μg/0.1 mL PBS *in vivo*	SUB	UCF	Exosomal miR-2 l-3p transfer, inhibit PTEN and SPRY I	(38)
		Human USCs	200 μg/0.1 mL PBS *in vivo*	SUB	ExoQuick	Promoting angiogenesis and activating PI3K-Akt signaling pathway via transferring DMBT1 protein	(39)
		Human AD-MSCs	200 μg/0.1 mL PBS *in vivo*	SUB	UCF	mmu_circ_0000250/miR-128-3p/SIRT1 axis	(40)
		Rat AD-MSCs	100 μg/0.2mL PBS *in vivo*	SUB	UCF	Targeting on miR124, stimulating the Wnt/β-catenin pathway	(41)
Diabetic peripheral neuropathy	Metabolic abnormalities, vascular disorders theory, protein glycosylation, immune factors, vitamin deficiency theory	Mouse BM-MSCs	1 × 10^9^ particle	IV	UCF	Abundant miRNAs, targeting the Toll-like receptor (TLR)4/NF-κβ signaling pathway	(42)
		Rat BM-MSCs	not reported	not reported	UCF	Exosomal miR-133b transfer	(44)
		Rat BM-MSCs	0.5 μg/2 μL	ICV	UCF	Enhancing oxidative stress, enhancing remover glutamate from the brain and maintain K^+^ balance	(43)
		Rat BM-MSCs	3 × 10^11^ particle	IV	ExoQuick	miR-9/ABCA1 pathway, anti-inflammatory	(19)
		Rat BM-MSCs	not reported	*ex vivo*	UCF	miR-146a-expressing exosome transfer, anti-inflammatory	(45)

BM, bone matTow; UC, umbilical cord; USC, urine-derived stem cells; AD, adipose tissue; MSC, mesenchymal stem cell; MB, menstrual blood; GG, gingival; IV, intravenous; RSI, renal subcapsular injection; SUB, subcutaneous; ICY, intracerebroventricularly; UCF, ultracentrifugation.

## Mesenchymal Stromal Cell Derived Small Extracellular Vesicles

Extracellular vesicles (EVs) refer to vesicle-like bodies with a double-layer membrane structure that fall off the cell membrane or are secreted by cells. They are widely present in various body fluids and cell supernatants, and stably carry some important signal molecules (46). According to MISEV 2018, they can be divided into 3 subgroups: small EVs (< 100 nm or < 200 nm), medium/large EVs (> 200 nm) (47). EVs are involved in cell communication, cell migration, angiogenesis, tumor cell growth and other processes. They act as new mediators of long-distance cell-to-cell communication and can transfer various biologically active molecules (such as encapsulated cytokines and genetic information) from their parental cells to distant target cells (47).

**Figure 1:**
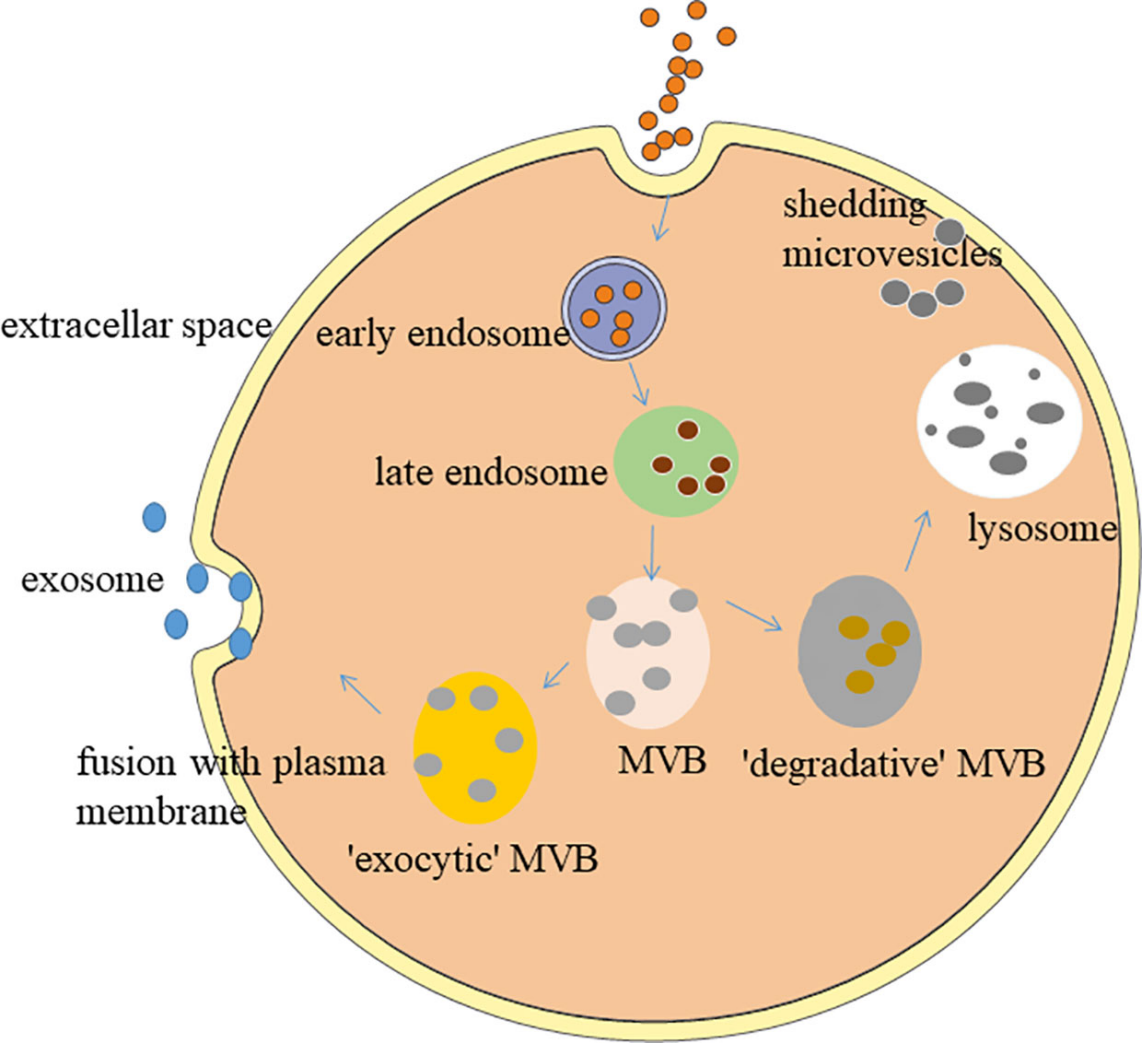
Biogenesis of small extracellular vesicles. The formation of sEVs originates from a series of regulation processes of “endocytosis-fusion-efflux”; that is, early endosomes formed after the plasma membrane of the cell is recessed. The envelope of early endosomes keeps invading and selectively accepts biologically active components, such as proteins, nucleic acids, and lipids in the cells, and eventually forms late endosomes. Late endosomes produce multiple intraluminal vesicles in the form of internal budding, which selectively receive cytosolic proteins, nucleic acids, or lipids to form multivesicular bodies (MVB). MVB are divided into exocytic-MVB and degradative-MVB. Exocytic-MVB release the vesicles in the MVB to the outside of the cell in the form of exocytosis through the plasma membrane fusion mediated by Rab27, and the released membranous vesicles are the sEVs. Degradative-MVB are degraded by fusion with lysosomes (50, 51).

Exosomes, the smallest EVs with the size range of 30 - 150 nm in diameter,have a bilayer structure and saucer-like morphology (48). In the full text of this article, we all use the exosomes command as sEVs. After fusion with the cell membrane, the contents of the sEVs are released into the extracellular matrix (49) as depicted in **Figure 1**. Almost all types of cells can secrete sEVs, mainly from body fluids, such as blood (19), urine (39), cerebrospinal fluid (52), saliva (53), and breast milk (54). sEVs apply their effects through targeting their cargos, such as nucleic acids (DNA, mRNA, miRNA, lncRNA and so on), lipids and proteins at the host cells, which leads to a shift in the behaviour of the recipient cells (55, 56). sEVs play an important role in various physiological and pathological processes, such as adipose metabolism, angiogenesis, inflammatory response, tissue regeneration, tumorigenesis, nerve regeneration, islet resistance, and immune regulation (57, 58). The current gold standard for isolating sEVs is ultracentrifugation (59, 60). The main molecular markers of exosomes include tetraspanin (CD9, CD63, CD81) and ESCRT proteins (TSG101, ALIX) (61–64). The methods for identifying sEVs mainly include the use of protein immunoassays. Western blot (WB) is used to detect molecular markers (65). Ultrastructure and particle size are measured by transmission electron microscopy (TEM) (66). Dynamic light scattering (DLS) or nanometer particle tracking analysis (NTA) detects particle size distribution (67).

MSCs can be isolated from various adult tissues such as bone marrow, umbilical cord, adipose, peripheral blood, liver, and gums (68). It is also favored for its advantages, such as easy collection, low immune rejection, and less ethical controversy (69). It has been used in the repair and regeneration treatment of various tissues and organs. Previous studies have shown that by MSCs being transplanted into damaged tissues and differentiated to replace damaged cells, systemic administration of MSCs can prevent the occurrence of T1DM. However, the proportion of transplanted MSCs reaching the damaged tissue was less than 1%, and most of them were trapped in organs such as liver and lung (70). Even if the transplanted MSCs had tissue repair effects, the transplantation and differentiation of MSCs in the damaged parts was low and short-lived (71), suggesting that MSCs exert the greatest effect through the secretion media. Simple MSCs transplantation may also have the risk of embolism, abnormal differentiation and tumor formation, these factors limit the use of MSCs (72). MSCs are recruited to the damaged tissue and secretes cytokines through paracrine action to change the microenvironment and induce the repair of the tissue structure and function of the damaged organ. In addition to several common modes (endocrine, paracrine, chemical synapse communication between neuron and target cell, autocrine, contact dependent communication between cells), there are other modes of information communication between cells through exosomes. Due to the vesicle structure of sEVs, the way of information exchange between cells is different from the typical mode mentioned above. There are three main ways for sEVs to participate in information exchange between cells, including the direct action of signal molecules on the membrane surface, intracellular regulation of content during membrane fusion and regulation of release of bioactive components. The discovery of sEVs makes the information exchange between cells more precise and comprehensive. The discovery of sEVs reveals the RNA intercellular transfer pathway existing in the body itself, which is expected to become an ideal gene therapy vector in the field of gene therapy due to its safe and effective targeted transport capacity. (73). MSCs are prolific sEVs secretors that shed a higher dose of sEVs compared to other cell types (74, 75). Indeed, most *in vitro* studies have directed their focus on sEVs derived from MSC subtypes, including adipose-derived stem cells (ADSCs), umbilical cord mesenchymal stem cells (UC-MSCs), bone marrow-derived mesenchymal stem cells (BM-MSCs), and others. The amount of sEVs released from MSCs is strongly associated with their proliferation rate (75). MSC-sEVs seem to be particularly beneficial in enhancing the recovery of various disease models. A recent study found that MSC-sEVs have tissue repair and reconstruction functions similar to MSCs. MSC-sEVs act on target cells such as β-cells, adipose cells, liver cells, macrophages, and skeletal muscle cells, by carrying specific miRNAs, proteins and other substances, thereby participating in the occurrence and development of IR and diabetes. As a new research hotspot, compared with MSCs, MSC-sEVs have the following advantages (76–78): (1) MSC-sEVs do not have the same issues as human cell transplantation, so the risk of immune rejection and aneuploidy is lower, and the safety is higher. There are also no ethical issues. (2) MSC-sEVs is stable in nature, can be stored for a long time at -20 °C, are easy to manage and control, and the type and quantity of its contents can be changed artificially. Also, its contents are not easily degraded (79, 80). (3) MSC-sEVs are small in size and will not block capillaries as easily as MSCs. The dosage of MSC-sEVs can be adjusted as needed. Many of the proteins found in MSC-sEVs are enzymes, which are milder than drugs and may reduce the risk of overdose or under-dose (81, 82). (4) MSC-sEVs have a more direct effect and can target specific organs and damaged parts. MSC-sEVs can cross the blood-brain barrier due to its own structural characteristics and have higher histocompatibility. (5) The production cost of MSC-sEVs is controllable, and MSC-sEVs can be conveniently transported and stored.

## Study on the Application of Small Extracellular Vesicles in the Diagnosis of Diabetes

### Small Extracellular Vesicles -Related miRNAs

Increasing blood glucose is the main criterion for diagnosing diabetes. Although the detection method is simple and cost-effective, it cannot provide relevant information about the patient’s pathogenesis or disease progression. During the onset and development of diabetes, the morphology and number of circulating sEVs change with the individual’s physiological or pathological conditions, suggesting that it may be used as a new marker (83). Studies have shown that sEVs can be used as carriers to transport miRNAs or other RNAs to neighbouring cells through the binding of receptors and ligands, and as biomarkers to indicate physiological or pathological changes in tissues or organs. miRNA is a type of small single-stranded RNA with a length of 18-25 nucleotides. It can mediate post-transcriptional gene silencing by binding to the 3’-untranslated region or open reading frame region of the target mRNA (84). It plays an important regulatory role in the process of differentiation, migration, and disease occurrence and development. Exosomal miRNA is protected from RNase degradation by the phosphatidic acid molecular layer, which is conducive to the isolation, extraction and storage of exosomal miRNA (85). Garcia-Contreras et al. (86) performed exosomal miRNA chip detection on plasma samples of 12 patients in the T1DM group and 12 healthy person in the control group, and the results demonstrated that there were significant differences between the two groups of 7 miRNAs, and the up-regulated miR-16- 5p, miR-302d-3p, miR-378e, miR-570-3p, miR-574-5p, and miR-579; miR-25-3p were down-regulated. It was verified by RT-qPCR that miR-16-5p and miR-574-5p in the control group were significantly higher than in the T1DM group. This study revealed for the first time that miRNA isolated from patient plasma sEVs was expected to be a potential biomarker for the diagnosis of T1DM. Glutamate decarboxylase-65 antibody, insulin antigen-512 and insulin antibody are the main autoantibodies in patients with T1DM. The sEVs containing autoantibody-positive miRNAs or proteins secreted by pancreatic β-cells can be used for the diagnosis of T1DM potential markers (86, 87). In addition, circulating sEVs in the body fluids of diabetic patients show higher procoagulant activity (88). Compared with the normal healthy group, whether they have microvascular complications or uncomplicated, all patients with T1DM have a significantly higher sEVs level. It can be used as one of the diagnostic basis (89). Meanwhile, Katayama et al. found that exosome-derived extracellular miR-20b-5p, a highly abundant exoRNA in patients with T2DM, is a circulating biomarker associated with T2DM that plays an intracellular role in modulating insulin-stimulated glucose metabolism *via* AKT signalling (90). Moreover, in peripheral blood microvesicles, among 104 miRNAs in EVs, miR-320-3p is highly expressed in microvesicles of plasma (2.637-fold more than in blood cells), as well as, miR-320-3p has also been associated with the regulation of Glucose-Induced Gene expression in T2DM (91). During the occurrence and development of diabetes, the content or number of sEVs will change abnormally, which suggests the possibility for using sEVs as new markers. In addition, quantitative and stoichiometric analyses of miRNAs content in sEVs highlight the lack of reliable natural sources for miRNA-loaded particles, which necessitates the need for custom sEVs or nanoparticles to efficiently deliver miRNAs closely related to immunity, metabolism, and epigenetics in target cells. However, loading extracellular mature miRNAs into recipient cells comes at a cost, as it at least blocks the dynamic localization of miRNAs in the nucleoli, or leads to inefficient miRNAs delivery due to rapid exonuclease recovery. All of this work requires the design of new bionic vectors and *in vivo* assessment of miRNA function when delivered by natural or bionic nanoparticles to control metabolic diseases from infancy to adulthood.

### Small Extracellular Vesicles-Related Protein

The latest research shows that C-megalin in urinary sEVs is positively correlated with the severity of DN (92). With the increase in urinary albumin, the level of C-megalin in urinary sEVs also increases, which is expected to become a diagnostic marker for DN. Aquaporins (AQPs) are a class of transmembrane proteins that are highly selective to water and have important physiological functions in regulating water metabolism. Polyuria is an early clinical symptom of diabetes. The levels of AQP2 and AQP5 in the urinary sEVs of DN patients are positively correlated with the histological grade of DN (93), indicating that AQPs of sEVs may become biomarkers for early diagnosis and monitoring of DN.

## The Role of MSC-sEVs in Diabetes

### The Improvement of Pancreatic β-Cell Function

MSC-sEVs improve the function of pancreatic β-cells, which may be one of its mechanisms of treatment of diabetes. After transplantation, MSC-sEVs can specifically chemoattract and migrate to the damaged islets to promote the proliferation of β-cells in the damaged islets, so as to repair and regenerate β-cells and inhibit β-cell apoptosis. Sabry et al. (94) found that injecting MSC-sEVs into streptozotocin (STZ) -induced diabetic rats had a better hypoglycaemic effect and faster effect than MSCs themselves. In the MSC-sEVs group, blood glucose levels decreased, plasma insulin levels increased, islet cell regeneration was enhanced, the number and size of islets increased, fibrosis and inflammation decreased, and the islet regeneration genes insulin, Pdx1, Smad2, Smad3, and Tgf-b were all significantly up-regulated. Another study found that sEVs isolation from menstrual blood-derived-MSCs through homing to the pancreas and pancreatic and duodenal homeobox 1 pathway enhanced the STZ-induced wistar rat β-cell quality and insulin production (95). Chen et al. (96) used the mouse β-cell line βTC-6 and found that the expression of apoptosis-related proteins cleaved caspase 3 and poly ADP-ribose polymerase (PARP) were up-regulated under hypoxia. MSC-sEVs with miRNA-21 reduced endoplasmic reticulum stress-related protein (GRP78, GRP94, p-eIF2α and CHOP) expression and inhibited of p38/MAPK phosphorylation, thereby protecting β-cells from hypoxia-induced apoptosis. MSC-sEVs are as effective as parental MSCs in improving the survival rate and function of islet cells (97). This cytoprotective effect may be mediated by vascular endothelial growth factor (VEGF) in MSC-sEVs (97). Another study by Kordelas et al. (98) demonstrated that MSC-sEVs can also help the angiogenesis and survival of transplanted pancreatic islets, improving the efficiency and success rate of the treatment. Overall, MSC-sEVs can improve the survival and function of the coated islets and benefit diabetic patients.

### Amelioration of Insulin Resistance in Peripheral Target Tissues

#### Regulation of Autophagy

Autophagy is an important regulatory pathway for maintaining cell homeostasis, and maintains normal cell function by affecting the degradation of intracellular substances. The dysregulation of autophagy-related mechanisms after diabetes can lead to a decrease in the number of pancreatic β-cells and dysfunction, resulting in a decrease in insulin secretion (99). The latest study found that MSC-sEVs can promote liver glycolysis, glycogen storage and lipolysis of 0.25mM palmitic acid (PA)-treated LO2 cells and reduce gluconeogenesis (100). They found that the AMPK signaling pathway was activated and induced autophagy in T2DM rats and PA-treated LO2 cells. The formation of autophagosomes in the MSC-sEVs group increased, and the autophagy marker proteins BECN1 and MAP 1LC3B increased. Furthermore, the autophagy inhibitor 3-methyladenine significantly reduced the effect of MSC-sEVs on glucose and lipid metabolism in T2DM rats.

#### Mechanism of Insulin Resistance in Peripheral Target Tissues

Sun et al. (101) established a T2DM rat model with high-fat diet (HFD) and STZ induction. Through the results of oral glucose tolerance tests (OGTTs), peritoneal insulin tolerance tests (IPITTs), IR index (HOMA-IR) and serum insulin tests, they found that intravenous injection of sEVs from MSCs (hucMSC-sEVs) can effectively alleviate hyperglycemia in T2DM rats. In T2DM rats, hucMSC-sEVs restored the phosphorylation of insulin receptor substrate 1 (IRS-1) and protein kinase B (Akt), and promoted the expression and membrane translocation of glucose transporter 4 (GLUT4) in muscles. The expression level of glycogen synthesis related protein p-GSK3β and glycogen synthase rises, increasing the storage of liver glycogen to maintain glucose homeostasis. At the same time, hucMSC-sEVs inhibited STZ-induced β-cell apoptosis and restored the insulin secretion function of T2DM. The study of Su et al. (102) effectively explained a clinical phenomenon, which is “Why do the elderly often develop IR?” They extracted the sEVs released by BM-MSCs from young and aged mice. They found that the highly enriched miR-29b-3p sEVs released by BM-MSCs can be absorbed by adipocytes, cardiomyocytes and hepatocytes, thereby producing IR *in vivo* and *in vitro*. Their research further found that miR-29b-3p could directly target SIRT1. Interestingly, they utilized an aptamer-mediated nanocomplex delivery system, which can specifically target BM-MSCs. Down-regulating/up-regulating the level of miR-29b-3p in BM-MSCs-derived sEVs could significantly ameliorate/increase IR in elderly mice.

#### Anti-Inflammatory and Immune Regulation Mechanism

**Figure 2:**
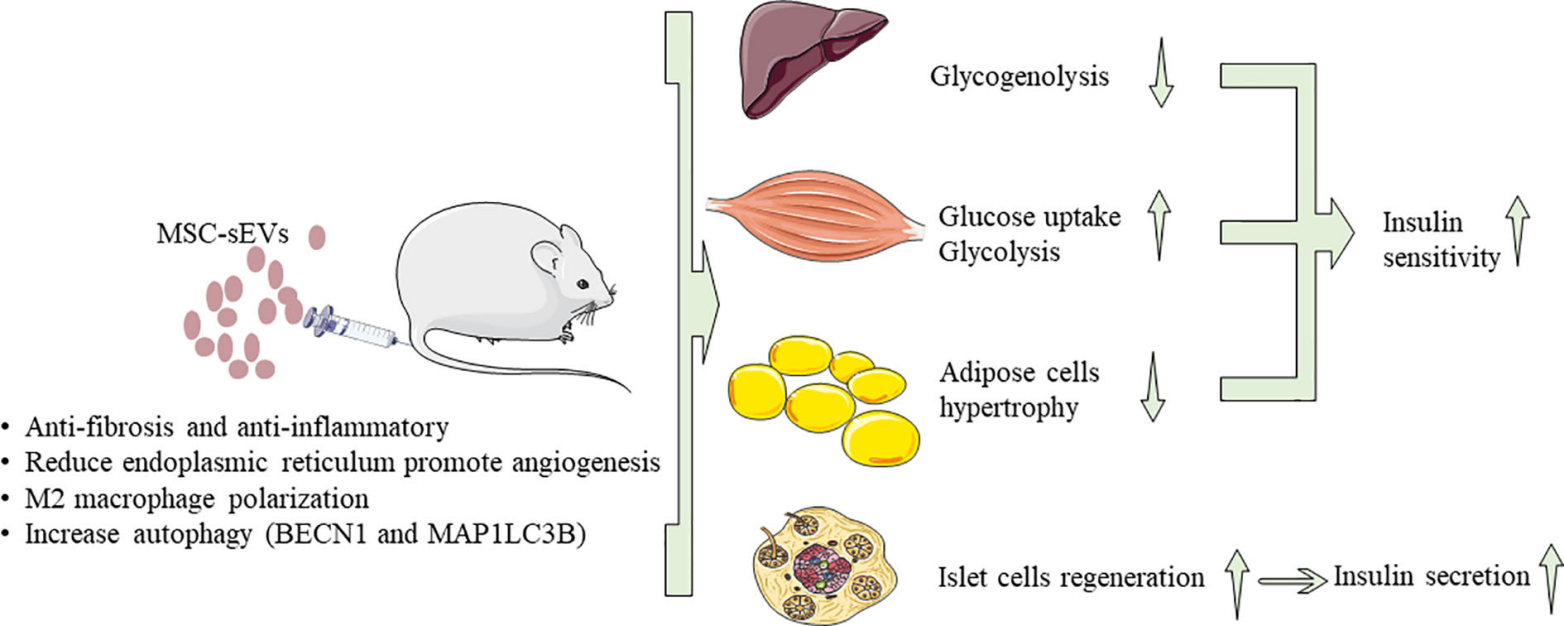
The approach of MSC-sEVs to treat diabetes. MSC-sEVs down regulate blood glucose through reverse insulin resistance in insulin target tissues (liver, muscle, adipose tissue) and relieve islet β cells destruction.

Immunity imbalance is one of the key factors in the pathogenesis of diabetes. Generally, inflammatory cells secrete pro-inflammatory cytokines, such as tumor necrosis factor-α (TNF-α) and interleukin 6 (lL-6) (101, 103), which are the main causes of IR in chronic inflammatory tissues. Therefore, timely and effective ameliorate the body’s microenvironment and regulation of immune response through MSC-sEVs is one of the important directions and strategies for diabetes treatment. As we all know, T1DM is an autoimmune disease characterized by permanent destruction of pancreatic β-cells mediated by T cells (104). Recent studies found that the autoimmune response or immune imbalance was closely related to IR and the progressive decline of pancreatic β-cell function during the pathogenesis of T2DM (105). In addition, the incidence of diabetes-related complications is also affected by autoimmune reactions (106). Sun et al. (101) found that, in the STZ-induced rat diabetes model, hucMSC-sEVs injected *via* the tail vein inhibited the secretion of pro-inflammatory cytokine TNF-α to reverse T2DM IR and indirectly increased the insulin/AKT signaling pathway activation. Zhao et al. (107) introduced ADSCsEVs into mice fed with HFD-Fed, which can significantly reduce systemic IR caused by obesity, attenuation of dsylipiemia, inhibit fat cell hypertrophy. By measuring the area under the glucose tolerance test curve, it was observed that ADSCsEVs can increase the effect of insulin by 27.8%. The transfer of ADSC-sEVs carrying active STAT3 to activate arginase-1 from adipose-derived mesenchymal stem cells to macrophages induces polarization of M2 macrophages and attenuation of WAT inflammation in HFD-Fed Mice. M2 macrophages induced by ADSC–sEVs can express high levels of tyrosine hydroxylase. Also ADSC–sEVs can drive the expression of Arg-1 by transporting STAT3 to promote ADSCs proliferation and lactate production. The Fas/FasL pathway plays an important role in β-cell apoptosis in T1DM, especially under high glucose conditions (108). Human BM-MSCs and their sEVs can deliver siFas and anti-miR-375 together to inhibit the early apoptosis of transplanted human islets (109). Under inflammatory cytokine treatment, after simultaneously silencing Fas and miR-375, pancreatic islet cell apoptosis was markedly inhibited, and insulin release was enhanced. In the humanized NOD scid gamma mouse model, intravenous injection of BM-MSCs and peripheral blood mononuclear cells (PBMC) co-cultured sEVs can further suppress the immune response by inhibiting the proliferation of PBMC and enhancing the function of regulatory T cells (Treg). BM-MSCs and derived sEVs may be an effective method to improve islet function under inflammation as showed in **Figure 2**.

## The Role of MSC-sEVs in the Complications of Diabetes

### Microvascular Disease

Microvessels refer to capillaries and microvessels between tiny arteries and tiny veins with a lumen diameter of less than 100 µm. Microangiopathy is a specific complication of diabetes. Its typical changes are microcirculation disorder and thickening of the microvascular basement membrane. The main risk factors include long duration of diabetes, poor blood sugar control, high blood pressure, dyslipidemia, smoking, and IR. Microvascular disease mainly manifests in the retina, kidney, nerve, and myocardial tissue, among which DN and diabetic retinopathy (DR) are particularly important. Fortunately, MSC-sEVs has been found to have a positive therapeutic effect in these microvascular diseases.

#### Repair Function of MSC-sEVs in Diabetic Nephropathy

DN occurs in 20% to 40% of diabetic patients (110). DN has become one of the main causes of end-stage renal disease. Diabetes with long-term hyperglycemia infiltration and micro-inflammatory environment can cause substantial kidney damage. Its clinical feature is proteinuria, and its final pathological manifestation is renal fibrosis. Studies have pointed out that sEVs not only participate in the early diagnosis of DN, but also have the ability to repair kidney tissue. Ebrahim et al. (11) found that STZ-induced diabetes rat after tail vein injection of MSC-sEVs can ameliorate kidney injury. A kidney biopsy showed that glomerular and tubular collagen fibre deposition was reduced, glomerular basement membrane thickened and foot process fusion was reduced. Many autophagosomes can be seen in the cytoplasm. RT-qPCR and WB showed that the expression of mTOR was reduced, and the expression of autophagy-related proteins LC3II and Beclin-1 increased. It may be possible to up-regulate the expression of LC3II and Beclin-1 by inhibiting the mTOR signalling pathway to enhance autophagy. In turn, it reduced podocyte apoptosis and collagen fibre deposition and exerted renal protection. In addition, ADSC-sEVs also have a renal protective effect (27). After the tail vein injection of ADSC-sEVs in mice, blood urea nitrogen, serum creatinine, and urine protein decreased, and podocyte apoptosis decreased. The addition of ADSC-sEVs co-cultivation can effectively alleviate the limitation of high glucose culture on the proliferation rate of MPC5 cells. The exosomal miR-486 reduced the expression of Smad1, inhibited the activation of p62/mTOR signalling pathway and caspase3, up-regulated the expression of LC3 and Beclin-1, enhanced autophagy, and reduced podocyte apoptosis to improve kidney injury in db/db mice. Exosomal miR-486 may be a key factor for stem cells to improve DN. MSC-sEVs protect the kidney by regulating autophagy-related factors, providing a new direction for the treatment of DN. Nagaishi et al. (28) found that the treatment of MSCs and MSCs sEVs can inhibit the abnormal infiltration and interstitial fibrosis of BMSCs in the kidney and inhibited inflammatory signals by inhibiting the p38-MAPK pathway and the overexpression of TGF-β. It reversed the severe damage to renal tubular epithelial cells (TECs) caused by high glucose stimulation, inhibited epithelial–mesenchymal transition (EMT), and reduced the damage caused by DN. In addition, in the diabetic rat model induced by STZ, the tail vein injection of human urine stem cell-derived small extracellular vesicles (USC-sEVs) can promote angiogenesis and cell survival by inhibiting the overexpression of caspase-3 protease and reduce podocyte apoptosis (29). Another study by Jiang et al. (18) demonstrated that USCs-sEVs could potentially reduce the urine volume and urinary microalbumin excretion, prevent podocyte and tubular epithelial cell apoptosis, suppress the caspase-3 overexpression, and increase glomerular endothelial cell proliferation in diabetic rats. Other related studies have also found that MSCs and its sEVs also delay the EMT of podocytes and renal tubular interstitial cells induced by high glucose and have anti-fibrotic effects on DN (31, 32). Overall, these findings provide the basis for the future application of MSC-sEVs as a new biological treatment of DN. However, its therapeutic value is currently limited to animal models. If MSC-sEVs treatment is to be applied into the clinic, further clinical trials are needed.

#### Repair Function of MSC-sEVs in Diabetic Retinopathy

Hyperglycemia can damage the basement membrane, endothelial cells and retinal perivascular cells of the retinal vascular system (111). Early treatment is to strictly control blood sugar and blood pressure to control disease risk factors, and later can be treated by laser photocoagulation, intraocular anti-VEGF, or glucocorticoid drugs (112). However, these treatments have certain complications and related problems, such as unstable efficacy. Therefore, the treatment of MSC-sEVs has become an alternative solution. At present, scholars generally believe that the occurrence of DR is related to retinal cell degeneration (including retinal cell apoptosis, glial cell dysfunction) and retinal microvascular dysfunction (retina no perfusion, changes in vascular permeability, and retinal neovascularization) (113). The results of Zhang et al. (25) showed that MSC-sEVs injection can alleviate the inflammatory response of diabetic rats or human retinal endothelial cells exposed to high glucose by down-regulating the levels of caspase-1, IL-1b, and IL-18. Compared with control MSC-sEVs, overexpression of miR-126 in MSC-sEVs significantly inhibited the HMGB1 signalling pathway in diabetic rats and inhibited inflammation. *In vitro*, miR-126 overexpression in MSC-sEVs significantly reduced HMGB1 expression and NLRP3 inflammasome activity induced by high glucose. Safwat et al. (24) showed that ADSC-sEVs reduce STZ-induced DR degeneration in rabbits by delivering micRNA-222 to retinal cells. Mathew et al. (23) explained that in the rat model, MSC-sEVs injected into the vitreous humor 24 h after retinal ischemia can significantly enhance functional recovery and reduce neuroinflammation and cell apoptosis. Generally speaking, MSC-sEVs have great potential as a biomaterial for neuroprotection and regeneration therapy of retinal diseases.

### Macrovascular Disease

The predisposing factors of atherosclerosis such as obesity, hypertension and dyslipidemia, often occur in people with diabetes (mainly T2DM) (114). The prevalence of atherosclerosis in diabetic patients is higher, and the onset is earlier. The progress is faster. Atherosclerosis mainly invades the aorta, coronary arteries, cerebral arteries, renal arteries and limb arteries, causing coronary heart disease, ischemic cerebrovascular disease, renal arteriosclerosis, and other diseases. Studies have pointed out that sEVs derived from BM-MSCs can treat brain cognitive dysfunction caused by diabetes. Diabetic vascular calcification (VC) is a common pathological basis in diabetic vascular disease, which is characterized by the deposition of calcium phosphate in the cardiovascular structure (115–118). Advanced glycation end products (AGEs) are the main cause of diabetes-related vascular complications, including diabetic vascular smooth muscle cells (VSMCs) calcification, which can cause VSMCs to calcify (119). The thioredoxin-interacting protein (TXNIP) is a member of the α-arrestin family of inhibitory proteins. In a high glucose state, TXNIP can bind to thioredoxin (Trx) and cause its inactivation and activity. The increase in oxygen species and the production of vascular inflammation are closely related to the production of AGEs (120). Wang et al. (26) found that MSC-sEVs contained high levels of miR-146a. When it was co-cultured with VSMCs pretreated AGE modified bovine serum albumin (AGE-BSA), it could be transferred into cells, by targeting to inhibit the production of TXNIP to protect Trx activity, and inhibit reactive oxygen species (ROS) to prevent AGE-BSA-induced calcification. Therefore, MSC-sEVs may be a potential therapeutic target for VC and play an important role in diabetic vascular disease.

### Repair Function of MSC-sEVs in Diabetic Central Nervous System Damage

Diabetic neuropathy is divided into central nervous system disease and peripheral nervous system disease. Central nervous system diseases include brain diseases and spinal cord diseases (121). The abnormal glucose metabolism caused by hyperglycemia damages nerve cells and slows down the conduction speed of brain cells, which can be measured by neuroelectrophysiological equipment. Many people with diabetes have worse memory, reaction speed, and thinking and cognitive abilities than people without diabetes (122). The conduction velocity of the spinal nerve is slowed down, and the patient has symmetry deep paresthesias in the lower limbs, such as loss of position sense, unstable walking, and dysuria. The main cause of cognitive impairment in diabetic patients may be damage to hippocampal neurons and astrocytes. In a study published by Nakano et al. (43) intravenous injection of MSC-sEVs easily spreads from blood vessels to the brain parenchyma. It was internalized by astrocytes and neurons, enhancing the ability of astrocytes to resist oxidative stress. At the same time, MSC-sEVs enhanced the ability of astrocytes to remove glutamate from the brain and maintain K+ balance, thereby promoting neuronal function, brain balance and synapse formation and improving cognitive impairment caused by diabetes. Local injection of BM-MSC-sEVs may be an effective drug for the treatment of cognitive impairment caused by diabetes. Xin et al. (44) found that MiR-133b in sEVs released after stroke by MSCs can transfer to astrocytes and regulate the gene expression of middle cerebral artery occlusion (MCAo) rats. miR-133b regulates the expression of Ras homolog gene family member A (RhoA) and connective tissue growth factor (CTGF), thereby promoting neurite remodelling and growth, and promoting the functional recovery of nerve cells. Venkat et al. (19) found that MSC-sEVs treatment of T2DM stroke can increase the expression of tight junction protein ZO-1, reduce blood-brain barrier leakage and bleeding, reduce body weight and reduce the expression of inflammatory factors (MMP-9 and MCP-1). At the same time, it promotes the remodelling of white matter marked by the increase in axon and myelin density to produce the therapeutic effect of nerve function recovery. The therapeutic effect induced by MSC-sEVs may be partly mediated by reducing the expression of miR-9 and up-regulating the ABCA1-IGFR1 pathway. Kubota et al. (45) further found that an enriched environment promoted the up-regulation of miR-146a secreted by endogenous BM-MSC-sEVs and down-regulation of IL-1 receptor-associated kinase 1 (IRAK1) expression, thereby inhibiting the NF-κB pathway and reducing the production of TNF-α, thereby exerting an anti-inflammatory effect on damaged astrocytes and preventing diabetes-induced cognitive impairment. In addition, some scholars have extracted sEVs derived from BM-MSCs of TIDM rats and BM-MSCs of normal rats and injected them into the brains of TIDM stroke rats (123). They found that the former has the ability to remodel the cerebral blood vessels and white matter. The test results showed that the former serum miR-145 expression decreased, while the miR-145 target gene adenosine triphosphate binding cassette transporter 1 and insulin-like long factor 1 receptor expression increased. Studies have further confirmed that the sEVs transfected and knocked out miR-145 affect the degree of nerve growth, indicating that miR-145 plays an important regulatory role in neuroprotection. MSC-sEVs carry a large number of proteins and nucleic acids that protect nerves and nutrient nerves, can regulate related molecular pathways, protect myelin sheath, reshape synapses, repair damaged neurons, and so on to promote neuron growth and functional recovery. The above research results proved that MSC-sEVs treatment was a powerful tool for central nervous system damage in diabetic patients.

### The Role of MSC-sEVs in Diabetic Peripheral Neuropathy and Autonomic Neuropathy

Diabetic peripheral neuropathy (DPN) is one of the main complications of diabetes and one of the important causes of the incidence and death of diabetes (124, 125). There is currently no effective treatment for this disease. Fan et al. (42) applied BM-MSC-sEVs to a DPN mouse model. The results showed that, after treatment with sEVs, the expression of TNF-α in nerve tissues was reduced, and the levels of TGF-β, IL-10, and Arg1 increased, indicating that exosome treatment reversed the increase in M1 type macrophages and the decrease in M2 type macrophages caused by diabetes, which is achieved by the polarization of macrophages M2 to reduce inflammation and improve neurovascular function. From these results we know that MSC-sEVs can reduce neurovascular dysfunction and improve the functional recovery of DPN mice by inhibiting the expression of pro-inflammatory genes.

Erectile dysfunction (ED) is a common comorbidity of male diabetes, and its pathogenesis may be caused by dysregulation of corpus cavernosum smooth muscle cells (CCSMCs). According to epidemiological data, about 50% of diabetic male patients develop erectile dysfunction within 10 years after diagnosis (126). Considering the low efficacy of oral phosphodiesterase type 5 inhibitors (PDE5i) in these patients (127, 128), MSC-sEVs therapy is an attractive tool for the treatment of diabetic ED (DED). Zhu et al. (21) showed that ADSC-sEVs contains some pro-angiogenic microRNA (miR-126, miR-130a, and miR-132) and an anti-fibrotic microRNA family (miR-let7b and miR-let7c). ADSC-sEVs have pro-angiogenic properties *in vitro. In vivo* ADSC-SEVs can induce endothelial cell proliferation, reduce cavernous fibrosis, and restore erectile function. Wang et al. (20) also found that ADSC-sEVs promoted neurovascular function by delivering corin and inhibited the expression of inflammatory factors to restore erectile function in diabetic rats. Huo et al. (22) explained that miR-21-5p delivered by MSC-sEVs can inhibit the expression of PDCD4 in T1DM rats, thereby stimulating the proliferation of corpus caverno-sum smooth muscle cells (CCSMCs), inhibiting CCSMCs apoptosis, and improving DED. These findings may provide new insights into the role of MSC-sEVs in the innovative treatment of DED.

### The Role of MSC-sEVs in Diabetic Foot Ulcers and Diabetic Skin Damage

Diabetic foot ulcers (DFU) is a serious complication of diabetes. Although people are increasingly aware of its pathophysiology and cellular and molecular responses, the reason for this pessimistic situation is the lack of effective treatments. DFU is mainly caused by ischemic, neurological, or combined neuroischemic abnormalities (129). It is a slow-healing deep chronic wound and microvascular obstruction. In recent years, multiple studies have reported the potential of MSC-sEVs to treat lower extremity ischemia and ulcers caused by diabetes. MSC-sEVs show stupendous therapeutic potential in the immune regulation and angiogenesis stage of DFU. In the immune regulation stage, MSC-sEVs could secrete miR124a/125b (130) to reduce inflammation, produce leb-7b (36) to regulates macrophage polarization, and secrete miR21 (38, 131, 132) to modulate dendritic cell differentiation. In the angiogenesis stage, MSC-sEVs have the ability to produce NRF2 (37), mmu_circ_0000250 (40), DMBT1 (39), lncRNA H19 (133), OxOband (41), miR126 (134, 135), miR23 (136), and miR21 (38, 131, 132) to promote the process of angiogenesis, granulation tissue formation, and re-epithelialization. The detailed summary of this part can be found in the review by An et al. (137). The exosome-derived miRNA and protein can be better protected by the exosomal membrane structure to avoid degradation, which not only helps to open up new targets for the early treatment of DFU, but also delays or even reverses the disease caused by the DFU process.

**Figure 3:**
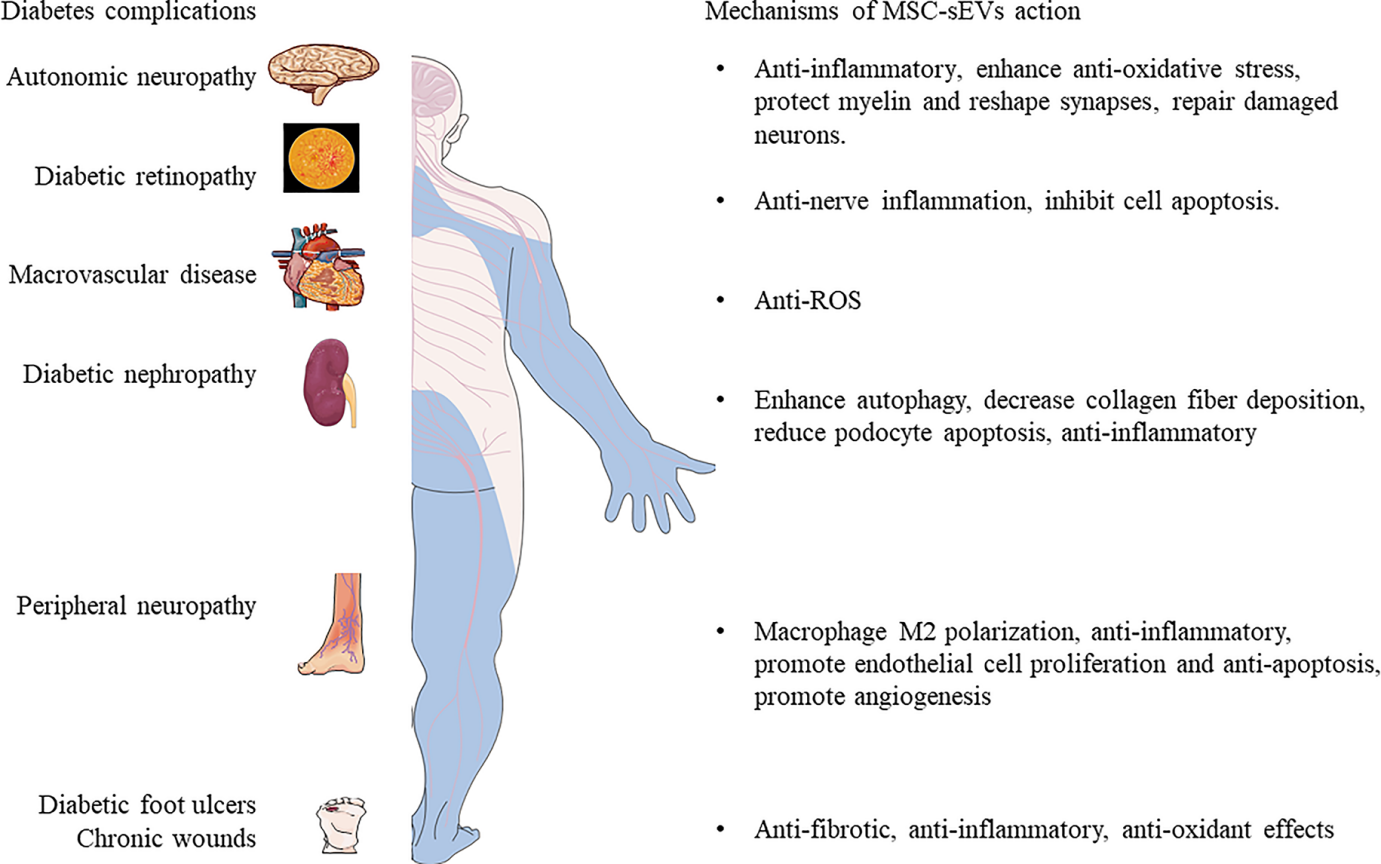
The mechanisms of MSC-sEVs in the treatment of complex diabetic complications.

Poor healing of diabetic wounds can increase the risk of gangrene, amputation and even death. The main reasons for this complication include hypoxia, impaired angiogenesis, reactive oxygen species injury, and neuropathy (37, 138). At present, the main methods for treating diabetic chronic skin damage are debridement and dressing, but these treatment methods do not bring satisfactory results (139). Studies have shown that MSC-sEVs can carry a variety of anti-inflammatory factors and growth factors, which can regulate the immune response and inflammation (140, 141), promote wound angiogenesis (142, 143), accelerate the proliferation and regeneration of skin cells (142), and activate the collagen secretion of fibroblasts (144), eventually promoting the re-epithelialization of the skin wound. ADSC-sEVs exert an immunosuppressive effect by reducing the secretion of IFN-α, thereby inhibiting the activation of T cells (145). Li et al. (37) found that ADSC-sEVs inhibited the production of ROS and inflammatory factors through the overexpression of Nrf2 in a high glucose environment and prevented the senescence of endothelial cells. In addition, a new method to promote the healing of diabetic wounds has emerged: Shi et al.’s study in diabetic rat skin defect models showed that the combination of sEVs derived from gingival MSCs and chitosan/silk hydrogel can promote the regeneration of extracellular matrix (35). Epithelialization, deposition and remodelling promote angiogenesis and inward growth of neurons, thereby effectively promoting the healing of ulcer wounds in diabetic rats. ADSC-sEVs riched in miRNA-125a and miRNA-31, which can be transferred to vascular endothelial cells to stimulate proliferation, and promote angiogenesis (142, 143). At the same time, ADSC-sEVs can inhibit the expression of angiogenesis inhibitor (DLL4) and the anti-angiogenesis gene HIF1 in vascular endothelial cells, thereby promoting the migration of vascular endothelial cells and enhancing angiogenesis. In the early stage, ADSC-sEVs promote collagen remodelling by synthesizing type I and III collagen and, in the late stage reduce scar formation by inhibiting collagen formation (144). In addition, ADSC-sEVs stimulate the reconstruction of the extracellular matrix by regulating the differentiation and gene expression of fibroblasts, thereby promoting wound healing. Wang et al. (146) found that ADSC-sEVs increased the ratio of transforming growth factor-β3 (TGF-β3) to TGF-β1 *in vivo*. ADSC-sEVs also increased the expression of MMP3 in skin dermal fibroblasts, which was beneficial to the remodelling of the extracellular matrix (ECM) to reduce scar formation. These evidences prove that MSC-sEVs or its combination with new materials have strong therapeutic potential as depicted in **Figure 3**.

## Pharmaceutical Development of MSC-sEVs-Based Therapeutics for Diabetes Diseases

With such promising preclinical findings in various types of disease models, investigators are now tasked with developing safe, feasible and reproducible MSC-sEVs-based therapies. Currently, 93 clinical trials involving exosomes are listed in www.clinicaltrials.gov. The majority of these trials focus on the use of sEVs from several body fluids as early diagnostic tools to predict the outcome of various treatments. MSC-sEVs have been shown in preclinical studies to be safe and scalable to large, clinically relevant doses (147).To date, several clinical applications of MSC-sEVs have been reported (148). A published study demonstrated that increasing dosages of MSC-sEVs in a patient with severe therapy-refractory acute graft versus-host disease (GVHD), was well tolerated and led to a significant and sustainable improvement of symptoms, which remained stable for five months (98). Globally, at least one clinical trial of MSC-derived sEVs for the improvement of β-cell mass in T1DM patients has been reported (https://clinicaltrials.gov/ct2/show/NCT02138331?term=MSC+exosomes&draw=1&rank=1). The first phase I clinical trial was initiated in 2014 with the aim of evaluating the safety of UC-MSC-sEVs in 20 patients with type 1 diabetes. Patients received a systemic injection of sEVs (ranging between 40-180 nm, in a dose of the supernatant produced from (1.22-1.51) × 106/kg/IV) at day 0 and of microvesicles (ranging between 180-1000 nm, in a dose of the supernatant produced from (1.22-1.51) × 106/kg/IV) at day 7. At the end of study (3 months) the following parameters are being evaluated: liver functions tests, kidney functions tests, HbA1c, glucose tolerance test, fasting and 2 h postprandial blood glucose levels, C-peptide chain level, and calculated total daily insulin dose. However, the status of the trial is unknown. More studies of MSC-sEVs-based therapeutics will be initiated in the near future (https://www.clinicaltrials.gov; unique identifier: NCT02138331, NCT03437759, NCT03384433) (98, 149). However, there remain significant challenges to translating this therapy into the clinic.

## New Strategy for MSC-sEVs Treatment

The treatment of diabetes and its related complications is a huge economic burden for all countries in the world. At present, the most common way to diagnose diabetes is to measure blood glucose levels. However, before the blood sugar rises, there are potential vascular damage and decreased insulin sensitivity, so it is particularly important to find new alternative treatments. Numerous studies have shown that MSC-sEVs can mimic the biological functions of MSCs. But, MSCs mostly reside in the liver, spleen, and lungs, reaching less than 1% of the injured site. Most of the MSCs that reach the target tissue disappear after a few days, and the fate of most MSCs is anoikis or phagocytosis. Only a small amount of MSCs stayed at the injury site for a long time. In recent years, with the deepening of sEVs research, scholars have proposed sEVs as a new biomarker for early diagnosis of diabetes. Studies have confirmed that injection of therapeutic sEVs can maintain a lasting biological effect within 6 weeks, suggesting that sEVs can transmit lasting signal information between cells (150). Whether or not it is advisable to develop MSC-sEVs therapy since it seems that the miRNAs on their own have a therapeutic effect rather than the MSC-sEVs? Our answer is that it is necessary to develop the treatment of MSC-sEVs. Although current studies on the mechanism of MSC-sEVs have reported that miRNAs play a major role (151), miRNAs will eventually need a vector for gene therapy involving miRNAs (152). Loading of extracellular mature miRNA into recipient cells comes with a cost by at least impeding dynamic localization of miRNAs in nucleoli or inefficient miRNA delivery due to rapid recycling by exonucleases. All these works are calling for the design of new biomimetic vehicles. There have been some reports of genetic drugs/carrier, but they can be eaten up by the immune system and they’re not very stable, they have some immunogenicity (153). SEVs have a transport function and can increase the potency of miRNAs or drugs by modifying aptamer to specifically target tissues (67) and *in vivo* assessment of miRNA functionality when delivered by natural or biomimetic nanoparticles in order to control metabolic diseases. MSC-sEVs have great potential in cell-free therapy. This therapy is safer and easier to operate than cell therapy. It can circumvent potential tumorigenicity (154–156), untargeted tissue differentiation, undesired immune response in stem cell transplantation (157), low survival rate of transplanted cells (158), heterogeneity of clinical donors, *in vitro* expansion, cryopreservation methods, poor safety. Moreover, small-size EV preparations that are isolated using protocols including a filtration step through 0.22 µm membranes can be considered as sterile and do not require an additional sterilization step. These advantages make sEVs a safer and more effective alternative to traditional viral vectors (159), and sEVs can be designed as carriers for targeted delivery of molecular therapy (160). Because sEVs can transmit bioactive molecules between cells to affect the insulin sensitivity of target cells, using sEVs as therapeutic agents and carriers to enhance β-cell proliferation and repair also provides a new direction for the treatment of diabetes. With the development of targeted therapy technology, MSC-sEVs have shown great potential as a biomarker for the diagnosis and prognosis of diabetes and related complications (77). MSC-sEVs mediate a variety of signaling pathways in diabetes and its complications, transmit different messages between cells, and regulate pathological activities and physiological functions. Due to different physiological or pathological conditions, sEVs and their contents will undergo corresponding differential changes. If biomarkers are found in them, the detection of body fluid sEVs may become an effective early diagnosis and treatment method for diabetes and its complications. SEVs contain a variety of proteins and biological genetic materials, and sEVs can travel throughout the whole body and even penetrate the blood-brain barrier, so proteins and macromolecular genetic materials can be loaded in the sEVs. If the corresponding drugs are wrapped into the sEVs, and then targeted and absorbed by cells, they will become an effective means of precision treatment. MSC-sEVs will become an ideal solution for cell-free therapy in the field of regenerative medicine. Proteins and miRNAs that help tissue regeneration can be packaged into specially designed MSC-sEVs. The ligands expressed by these packaged MSC-sEVs help to attach to therapeutic targets. Theoretically, injection of these sEVs will allow the drug to be delivered directly to specific target cells. This therapy can be used in patients with diabetic nephropathy or central nervous system damage to stimulate cell regeneration. What’s more, it can also be used as a supportive treatment for islet transplant recipients, prolonging insulin independence and reducing patients’ dependence on immunosuppressive drugs. All the advantages for MSC-sEVs treatment by comparison to MSCs donor cells as showed in **Table 2**. But the long-term effectiveness and safety of this treatment must first be evaluated. Research on sEVs is still in its early stages. There is no comprehensive clinical trial using MSC-sEVs-based regenerative therapy. Research progress depends on a deeper understanding of sEVs formation and signal transduction mechanisms.

**Table 2:** The advantages for MSC-sEVs treatment by comparison to MSCs donor cells.

1. SEVs as drug carriers.
As a nanocarrier, sEVs have the advantages of being similar to cell membranes, small in size, negatively charged, avoiding phagocytosis, generating immune escape, long circulation time, and being able to penetrate deep tissues. High biocompatibility and low immunogenicity. More significant safety. SEVs have no adverse effects on the kidney and liver. Concentration, dosage and route are easier to control. SEVs have cell targeting ability. SEVs have the ability to cross biological barriers: sEVs can cross the body’s thick tissue barriers, such as the blood-brain barrier.
**2. SEVs are used for disease diagnosis.**
Richer sample formats: Almost all body fluid samples contain sEVs. Thanks to the protection of the phospholipid bilayer, the contents of the sEVs have better stability. Circumvent potential ethical issues and tumorigenicity. “Cell-free therapy” therapy is safer and easier to operate than cell therapy.
**3. SEVs are used for treatment.**
Low immunogenicity. Easy to store, no need to proliferate, easy to use quantitatively and to recruit from the damage. sEVs are stored at -20°C for 6 months, and stored at -80°C for a long time without losing their biochemical activity. It avoids the inconvenience of cryopreservation and recovery of MSCs, and can be used after dissolution, and the use time is easy to grasp. Mass production: sEVs can be enriched in a large amount in the culture medium. Controllable: The function of sEVs can be changed by changing the cell environment.
**4. Problems to be solved with sEVs.**
Efficient extraction technology (the extraction method and its complicated classification system hinder its application). The concentration of sEVs in the injured area after local injection of MSC-sEVs in animal experiments is unknown. The optimal concentration to promote tissue regeneration or immune regulation, and the half-life of sEVs also needs more in-depth research. The sEVs secreted by different cells or the same cell under different physiological conditions may be different, and the contents and mechanisms of sEVs need to be further studied. SEVs transport a variety of biomolecules, and how to regulate recipient cells in the body and change the state and fate of cells is still unknown. Separation schemes suitable for large-scale preparation, purification and storage. Standardization schemes for quantification, molecular and physical characterization. Clear quality control (QC) standards for clinical use: to ensure that the quality, safety and effectiveness of the sEVs products produced are guaranteed. For example, sEVs should be stored in isotonic buffer to prevent pH changes during storage and freeze-thaw cycles.

## Challenges

Unfortunately, few experimental studies have compared the efficacy of MSCs and MSC-sEVs in diabetes and its chronic complications. MSC-sEVs therapy prevents abnormal renal function in HFD- and STZ-diabetic mice, similar to MSCs therapy (28). However, MSC-sEVs in HFD-diabetic mice conferred anti-inflammatory and renoprotective effects exceeding those of their parent MSCs (28). The combination of MSCs and MSC-sEVs was more effective to either one alone inhibit further immune response of transplanted islets, suggesting additive effects (109). Both strategies supported the notion that MSC-sEVs recapitulate the salutary effects of MSCs.

Although MSC-sEVs are a good natural carrier, uncertainty remains regarding sEVs fate, safety, isolation, characterization and long-term effects, which might impose important limitations on their path to clinical translation. In translational clinical studies, people have not yet reached a consensus on the dose of sEVs, the quantification of MSC-sEVs is essential to understanding the basic biological relationship between MSC-sEVs and its parent cells and the underlying interpretation of MSC-sEVs signals. Currently, researchers use several different methods to quantify the dose of sEVs, protein concentration and NTA, tunable resistance pulse sensing (TRPS) and flow cytometry, which make it difficult to compare studies with each other (161). Therefore, to help inter-study comparison, we need multiple quantifications using various quantification tools. Next, the route of administration still requires further clarification. Therefore, it is necessary to further research and establish a uniform administration procedure for sEVs.

The storability of MSC-sEVs is an important aspect, both for basic research and for clinical applications. Currently, there is no standardized procedure for the storage of MSC-sEVs. Whether MSC-sEVs is suitable for storage at 4°C, -80°C, -196°C or other temperatures is no standardized procedure (162). It’s still impractical to always use fresh MSC-sEVs preparations. In addition, storage vials can also affect the quality of MSC-sEVs, because MSC-sEVs may accidentally and irreversibly combine with certain materials (163). Due to the lack of data to address the impact of storage time and drugs on the stability and effectiveness of MSC-sEVs, it is necessary to develop a customized agreement for MSC-sEVs.

At present, there are still a series of problems, such as complicated sEVs extraction processes, low purity, expensive reagent supplies and so on (77). It is essential to expand the production of MSC-sEVs to meet the needs of clinical research (164, 165). A standardized sEVs detection platform should be established as soon as possible to accelerate the clinical transformation of large sEVs and big data, and provide a new direction for the diagnosis and treatment of diabetes. Based on the complexity of the biological substances contained in sEVs, the specific mechanisms and signaling pathways of sEVs involved in diabetes coupling still need to be further explored. Therefore, it is necessary to further research and establish a uniform administration procedure for sEVs.

## Conclusion and Future Perspective

With a more complete understanding of the mechanisms driving sEVs formation, sEVs could be engineered as vectors for the targeted delivery of molecular therapies injection of these sEVs would, in theory, allow for the discriminate delivery of medicine directly into specific target cells. It could also be of benefit as a supportive treatment for pancreatic islet transplant recipients, lengthening insulin independence and reducing the patients’ dependence on immunosuppressive medication. With great perspective, MSC-sEVs therapy brings a bright future for diabetes treatment. Besides, sEVs from MSCs as well as pre-treatment of MSC-sEVs can be regarded as a key breakthrough to improve therapeutic efficiency.

In conclusion, MSC-sEVs is a therapeutic option for diabetes and its chronic complications in the future. However, long term studies are required to evaluate the efficacy and safety of sEVs therapy to find new and novel strategies for the treatment of diabetes; and further studies in humans are necessary to investigate the results on animal models. In the future, one can hope that sEVs therapy can be used in reduce the blood glucose, restore insulin sensitivity along with other complications treatments and promises a new therapeutic approach in clinical applications. In addition, with rapid advances in bioengineering and cell modification technologies, the next step in the field of sEVs will be the engineering or modification of exosome surfaces and contents, which may be more specific, extending its application to more complex medical fields.

## Author Contributions

L-QY: manuscript writing and approving final version of manuscript. F-X-ZL: study conduct, data analysis, and manuscript writing. XL, FX, S-KS, BG, L-ML, M-HZ, YW and Q-SX: data analysis. All authors: reviewed the manuscript. All authors contributed to the article and approved the submitted version.

## Funding

This work was supported by funding from the National Natural Science Foundation of China (Nos. 81770881 and 82070910). Key R &; D plan of Hunan Province (2020SK2078).

## Conflict of Interest

The authors declare that the research was conducted in the absence of any commercial or financial relationships that could be construed as a potential conflict of interest.

## Publisher’s Note

All claims expressed in this article are solely those of the authors and do not necessarily represent those of their affiliated organizations, or those of the publisher, the editors and the reviewers. Any product that may be evaluated in this article, or claim that may be made by its manufacturer, is not guaranteed or endorsed by the publisher.
